# Prior influenza virus infection alleviates an arbovirus encephalitis by reducing viral titer, inflammation, and cellular infiltrates in the central nervous system

**DOI:** 10.1128/jvi.02108-24

**Published:** 2025-01-16

**Authors:** Isabelle J. H. Foo, Brendon Y. Chua, So Young Chang, Xiaoxiao Jia, Alice van der Eerden, John K. Fazakerley, Katherine Kedzierska, Lukasz Kedzierski

**Affiliations:** 1 Department of Microbiology and Immunology, The University of Melbourne, The Peter Doherty Institute for Infection and Immunity 534133 , Melbourne, Victoria, Australia; 2 Department of Veterinary Biosciences, Faculty of Science, University of Melbourne 98487 , Melbourne, Victoria, Australia; St. Jude Children's Research Hospital, Memphis, Tennessee, USA

**Keywords:** influenza virus, arbovirus, encephalitis, central nervous system, CD8^+ ^T cells, innate immunity, inflammation, Semliki Forest virus

## Abstract

Respiratory and encephalitic virus infections represent a significant risk to public health globally. Detailed investigations of immunological responses and disease outcomes during sequential virus infections are rare. Here, we define the impact of influenza virus infection on a subsequent virus encephalitis. We used a model system in which mice were given influenza A virus (IAV) infection 8 days prior to Semliki Forest virus (SFV) infection (IAV→SFV). IAV infection clearly attenuated the subsequent SFV infection with reduced titers of infectious SFV and lower levels of cytokines and chemokines in the central nervous system (CNS). In contrast, the SFV viremia in both IAV→SFV and SFV-only mice was comparable. Increased type I interferon (IFN) levels in the CNS after IAV infection might have contributed to some level of protection towards SFV infection in the CNS, suggesting that early control of SFV replication in the CNS during IAV→SFV infection led to reduced adaptive response, given the lower number of CD8^+^ T cells recruited to the brain in IAV→SFV infection. In lungs, however, prior IAV infection elicited effector CD8^+^ T cells with highly activated CD38 and/or CD25 phenotypes, while SFV-only infection elicited distinct effector CD8^+^ T cells with increased frequencies of KLRG1 expression, a hallmark of short-lived effector T cells. Taken together, our findings demonstrate that prior IAV infection can confer protective immunity toward secondary SFV infection, confirmed by reduced disease severity and inflammatory immune responses in the brain. Our work provides important insights into therapies and vaccine regimens directed against unrelated pathogens.

**IMPORTANCE:**

Influenza viruses are medically important human pathogens that caused epidemics and pandemics throughout history. Conversely, encephalitic arthropod-borne virus (arboviral) diseases affect both humans and domestic animals, creating massive public health issues. Influenza viruses circulate globally while arboviruses dominate tropical regions. Given both influenza virus and encephalitic arboviruses, such as alphaviruses, circulate in many regions globally, co-infections are likely to occur. In addition, arthropod-borne neurotropic infections are generally mild or asymptomatic, hence are likely to be unnoticed as a risk factor during influenza infection. However, the consequences of such co-infections are unclear. Our recent study showed that alphavirus infection preceding Influenza A virus (IAV) infection negatively impacted immune responses to the influenza virus in mice. Here, we aim to investigate the immune responses when the order of sequential infection with IAV and alphavirus are swapped. Altogether, our findings will provide key insights to improved diagnostics, preventative vaccines, and antiviral therapies.

## INTRODUCTION

Infections with more than one virus in an individual are likely to occur frequently. While co-infections with chronic viruses such as HIV-1 and hepatitis viruses have been well studied (1–3), immune responses following acute viral co-infections are relatively understudied. Infections with influenza or dengue viruses impose a large burden on health and socioeconomic well-being globally. While there are reports of co-infection between influenza and dengue viruses (4–7), scarce data exist on how immune responses and disease outcomes are affected by co-infections with influenza and unrelated arboviruses.

Experimental evidence from other model systems suggests that the sequence of viral infections, the time interval between infections, and the route of infection can all affect the course of the disease (8). Mice infected simultaneously with both influenza A virus (IAV) and *Streptococcus pneumoniae* (*S. pneumoniae*) had a mortality of 50%. In contrast, mice infected with the IAV strain, PR8 (IAV-PR8), followed by pneumococcus 7 days later had 100% mortality; all mice died within 24 h of the secondary infection (9). In another mouse study compared to co-infection at the same time, rhinovirus (RV) infection, followed 2 days later by IAV-PR8, ameliorated disease, whereas IAV-PR8 infection followed 2 days later by RV, exacerbated disease (10).

We recently demonstrated in mice that, compared to IAV-only infection, prior infection with Semliki Forest virus (SFV) resulted in delayed IAV clearance, hypercytokinemia, and exacerbated lung pathology (11). SFV infection prior to IAV resulted in dendritic cell paralysis, poor proliferation of IAV-specific CD8^+^ T cells, and reduced IAV-specific memory CD8^+^ T cell formation and recall responses.

To investigate whether the order of these two infections matters and how reversing them affects immune responses, we have studied the reverse sequential co-infection with IAV preceding SFV and investigated how this affects immune responses to SFV. Thus, the main focus of our study was to determine whether IAV-specific immune responses established during an acute phase of IAV infection, affect SFV disease severity and accompanying SFV-specific immunity, either by exacerbation or alleviation of symptoms.

Compared to infection with SFV alone, prior infection with IAV had a protective effect. Titers of infectious virus (SFV) and brain inflammation were reduced in the co-infected mice and SFV-specific long-term memory CD8^+^ T cell pools were reduced in magnitude.

## RESULTS

### Prior infection with IAV leads to reduced levels of infectious SFV in the brain

**Fig 1:**
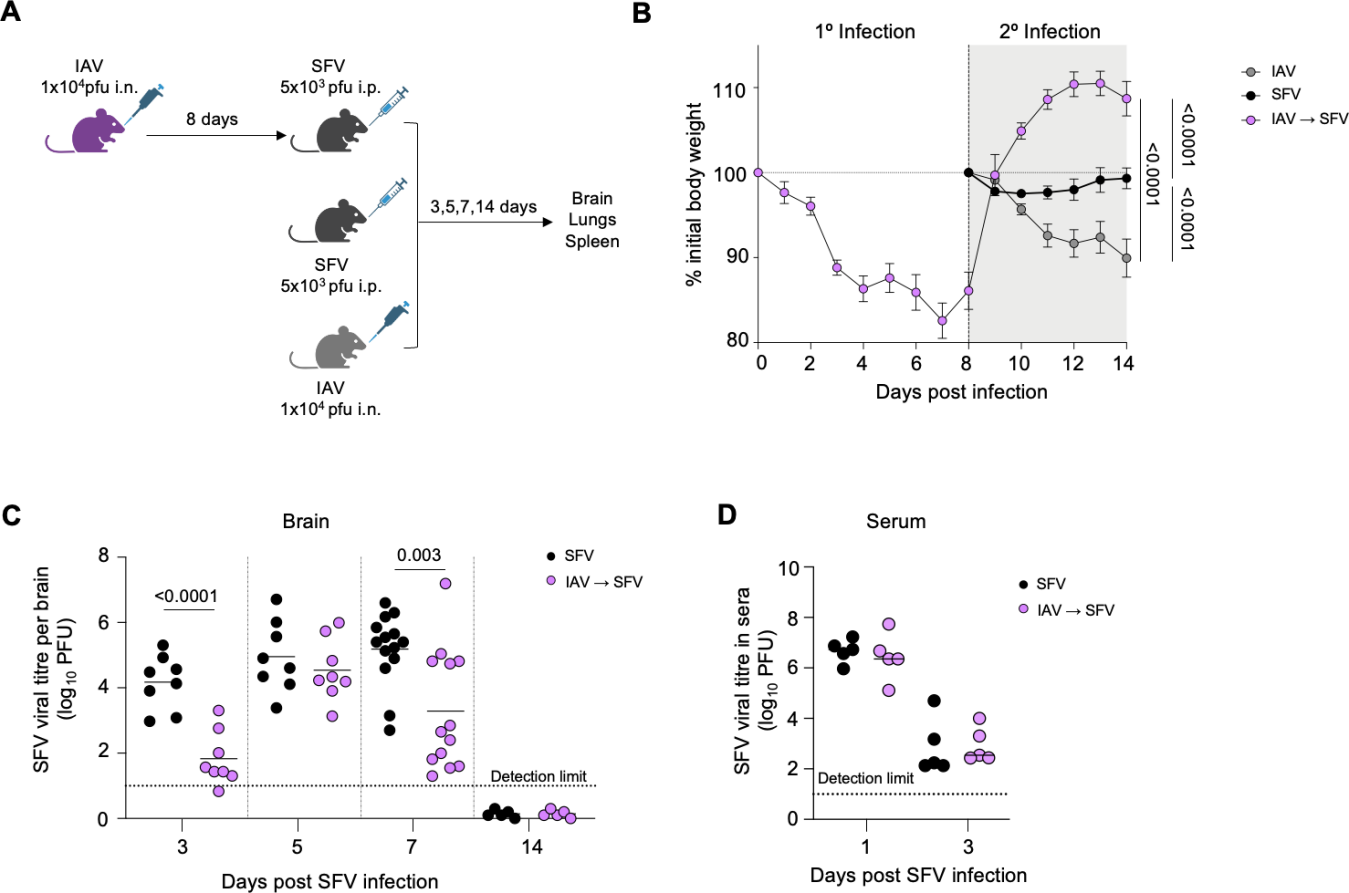
IAV→SFV sequential infection induces a protective response against SFV infection. (**A**) Mice were infected with 10^4^ pfu A/HKx31 (i.n.) followed 8 days later with 5 × 10^3^ pfu A7(74) SFV (i.p.), infected with SFV-only, or IAV-only. Brains, lungs, and spleens were harvested at various days post-infection (dpi). (**B**) Weight loss of SFV, IAV, and IAV→SFV infected mice was monitored for 14 days (*n* = 12–15, pooled data from three independent experiments, error bars represent SEM). Area under the curve (AUC) was determined by ordinary one-way ANOVA with Holm-Sidak’s multiple comparison test. (**C**) Brain SFV infectivity titers were determined by plaque assay on Vero cells. Each symbol denotes an individual mouse (*n* = 5–14, pooled data from three independent experiments). (**D**) Serum SFV titers were determined by plaque assay on Vero cells (*n* = 5, horizontal bars represent the group mean, pooled data from two independent experiments). Each symbol denotes an individual mouse. Significance was determined by unpaired two-tailed Student’s *t* test.

To study the impact of prior IAV infection on a subsequent SFV infection, adult C57BL/6 mice were infected with either 10^4^ pfu A/HKx31 (x31) IAV alone via the intranasal (i.n.) route, 5 × 10^3^ pfu A7(74) SFV alone via the intraperitoneal (i.p.) route, or 10^4^ pfu A/HKx31 (x31) IAV via the intranasal (i.n.) route followed by 5 × 10^3^ pfu A7(74) SFV i.p. on day 8 post-IAV infection (IAV→SFV sequential infection) (Fig. 1A). Weight loss following infection was recorded over 14 days. Mice infected with SFV-only showed up to 10% weight loss. Mice infected with IAV lost up to 20% of original body weight by 7 days post-infection (dpi), while IAV→SFV infected mice thereafter regained weight during the course of the subsequent SFV infection (Fig. 1B).

Levels of infectious SFV were determined at days 3, 5, 7, and 14 post-infection in brain following primary SFV (SFV-only) and secondary SFV (IAV→SFV) infection. On days 3 and 7, IAV→SFV mice had significantly lower SFV titers in the brain compared to SFV-only infection (Fig. 1C). To determine whether SFV replication was also affected systemically, virus titers were measured in the serum. There was no difference in systemic SFV infectious virus titer across time points (Fig. 1D), suggesting that reduced SFV replication in the co-infection group was confined to the brain microenvironment.

### Cytokine and chemokine levels are reduced in the brains and lungs of IAV→SFV infected mice

**Fig 2:**
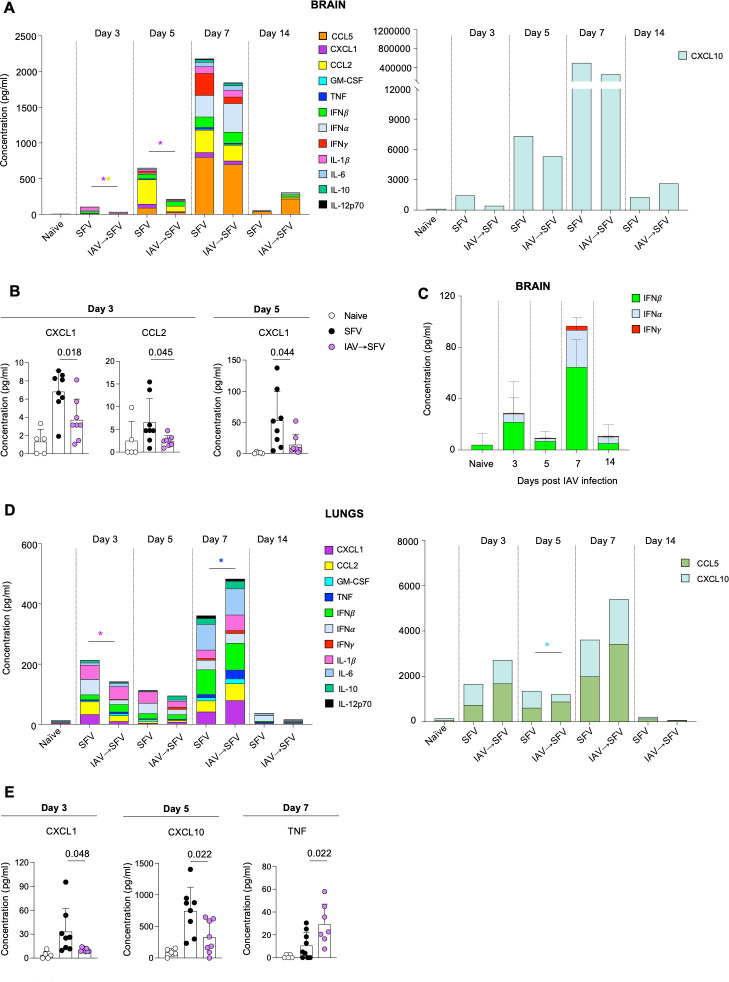
Cytokine and chemokine protein levels were lower in the brains and lungs of IAV→SFV infected mice. Brain and lung homogenates of naïve, IAV (brain only), SFV, and IAV→SFV infected mice were assayed by LEGENDplex to determine the cumulative levels of cytokines and chemokines at specific time points. (**A**) Cumulative concentrations of 12 cytokines and chemokines in the brains of naïve, SFV, and IAV→SFV mice, with CXCL10 plotted separately due to its higher levels (*n* = 5–9). (**B**) Comparison of significantly different chemokines in the brain. Each symbol denotes an individual mouse, pooled data from two independent experiments (*n* = 5–9). (**C**) Cumulative levels of type I and type II interferons in brain homogenates of naïve and IAV-only infected mice (*n* = 5). (**D**) Cumulative concentrations of 11 cytokines and chemokines in the lungs of naïve, SFV, and IAV→SFV mice, with CCL5 and CXCL10 plotted separately (*n* = 5–9). (**E**) Comparison of significantly different cytokines and chemokines in the lungs. Each symbol denotes an individual mouse, pooled data from two independent experiments (*n* = 5–9). Significance was determined by unpaired two-tailed Student’s *t* test.

To compare inflammation between SFV and IAV→SFV infections in brains and lungs, the levels of cytokines and chemokines were determined across the course of infection. In the brain, IAV→SFV mice had significantly lower levels of CXCL1 on days 3 and 5, and CCL2 on day 3 compared to SFV-only mice (Fig. 2A and B). Both chemokines facilitate chemotaxis and transmigration of leukocytes and lymphocytes to the brain in viral encephalitis (12–15). SFV replication is susceptible to interferons (IFNs), and IAV infection is known to induce the production of type I IFNs in infected lung epithelial cells and peripheral monocytes/macrophages (16–18). We speculated that IAV-only infection activates the production of IFN in the brain of IAV-infected animals, as this may contribute to the reduction of SFV replication in the brain of animals with a prior IAV infection. Thus, we investigated the level of cytokine and chemokines in the brain of IAV-only infection (Fig. S1A). We found that IAV infection induced IFNα/β in the brain peaking at 7 dpi, the day prior to SFV infection in our co-infection model system (Fig. 2C). Levels of most other cytokines and chemokines measured were similar in both SFV-only and IAV→SFV mice (Fig. 2A).

In the lungs, CXCL1 was significantly higher in the SFV-only infection at day 3, while CXCL10 was higher at 5 dpi (Fig. 2D and E). Conversely, TNF was higher on day 7 in IAV→SFV (Fig. 2D and E), while no significant differences were detected in the remaining cytokines/chemokines tested.

### Innate, humoral, and cellular immunity in IAV→SFV mice

To determine whether prior IAV infection modulates immune responses to SFV, we performed a broad analysis of myeloid cells, B cells, and T cells in brains, lungs, and spleens on day 7 following SFV infection. Day 7 post-infection allows for the investigation of peak cellular immunity in response to both viruses causing acute infection.

**Fig 3:**
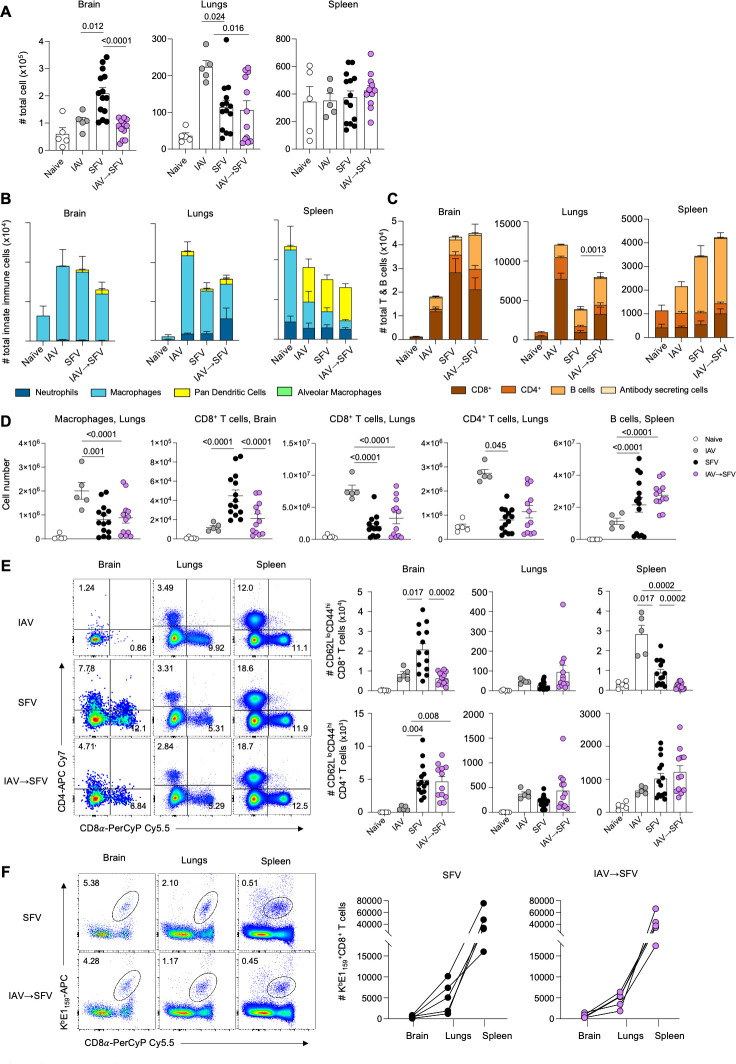
Analysis of leukocyte numbers and populations in the brains, lungs, and spleens of infected mice. (**A**) Total cell number of leukocytes in the brains, lungs, and spleens of naïve, IAV, SFV, and IAV→SFV infected mice at 7 dpi; (*n* = 5–14, error bars represent SEM). (**B**) Absolute numbers of neutrophils (CD11b^+^Ly6G^+^), macrophages (CD11b^+^CD64^+^F4/80^+^), alveolar macrophages (lungs only; CD64^+^CD11c^+^) and DCs (CD11c^+^) in the brains, lungs, and spleens of naïve, IAV, SFV, and IAV→SFV infected mice at 7 d.p.i. Mean data are shown for biological replicates (*n* = 5–14, error bars represent SEM). (**C**) Absolute numbers of B cells (B220^+^CD19^+^), antibody secreting cells (ASC, IgD^−^B220^lo^CD138^+^), CD4^+^ T cells and CD8^+^ T cells in the brains, lungs, and spleens of naïve, IAV, SFV, and IAV→SFV infected mice at 7 d.p.i. Mean data are shown for biological replicates (*n* = 12–14, error bars represent SEM). (**D**) Comparison of significantly different cell populations across different tissue types at 7 dpi (*n* = 5–14, bar and error bars represent mean ± SEM). (**E**) Absolute numbers of effector (CD44^+^CD62L^lo^) CD8^+^ (top row) and CD4^+^ (bottom row) T cells across different anatomical sites in naïve, IAV, SFV, and IAV→SFV infected mice. Representative FACS plots depict total CD4^+^ and CD8^+^ T cells for each group and tissues (*n* = 5–14, pooled data from three independent experiments, error bars represent SEM). (**F**) Absolute numbers of SFV-specific CD8^+^ T cells directed at K^b^E1_159_ epitope across different anatomical sites of SFV and IAV→SFV infected mice. Representative FACS plots shown for each group and tissues (*n* = 5, each line connecting dot represents a mouse). Significance was determined by ordinary one-way ANOVA, Tukey’s multiple comparison test. Data from 9 out of 15 SFV-only mice was used as a control in a previous publication, as indicated in source data (11).

We found that prior IAV infection led to a significant reduction of the cell numbers in the brains of SFV-infected mice, while the lungs and spleens had similar total cell count across SFV and IAV→SFV infected mice (Fig. 3A). IAV-only infection had lower total cell count than SFV-only infection in the brains, but higher cell count in the lungs compared to both SFV and IAV→SFV only mice (Fig. 3A). This is attributed to the fact that lungs are the primary site of infection of IAV infection.

Comparable numbers of neutrophils (CD11b^+^Ly6G^+^), macrophages (CD11b^+^CD64^+^), dendritic cells (DCs, MHC-II^+^CD11c^+^), and alveolar macrophages (lungs only; CD11c^+^CD64^+^) were detected across the SFV-only and IAV→SFV in the brains, lungs, and spleens. Similarly, we found comparable numbers of B cells (B220^+^CD19^+^), antibody-secreting cells (ASC, IgD^-^B220^lo^CD138^+^), and total CD4^+^ and CD8^+^ T cells across brains and spleens (Fig. 3B and C), despite having more leukocytes in the brain of SFV-only infected mice.

Analysis of specific immune cell types demonstrated that in the lungs, more total T and B cells were present in the IAV→SFV infection (Fig. 3C). Similarly, the total CD8^+^ T cell number was significantly higher in SFV-only infection compared to both IAV-only and IAV→SFV (Fig. 3D). Detailed analyses of cellular immune responses showed that IAV→SFV infected mice had lower numbers of effector (CD44^hi^CD62L^lo^) CD8^+^ T cells in the brains and spleens compared to IAV→SFV infected mice (Fig. 3E).

To further investigate immune responses toward SFV, we utilized previously defined (11) peptide-MHC-I tetramers (K^b^-E1_159-166_; TQFIFGPL; immunodominant CD8^+^ T cell SFV epitope in B6 mice) to identify SFV-specific effector CD8^+^ T cells. Overall, there was no difference in the number of SFV-specific CD8^+^ T cells across tissues and infection modes (Fig. 3F).

Altogether, our analysis indicates that, relative to SFV-only infection, IAV→SFV infection results in significantly reduced inflammation in the brain and increased numbers of CD8^+^ T cells, especially those with effector phenotype.

### Distinct activation phenotypes of effector CD8^+^ T cells within lungs of SFV and IAV→SFV infected mice

**Fig 4:**
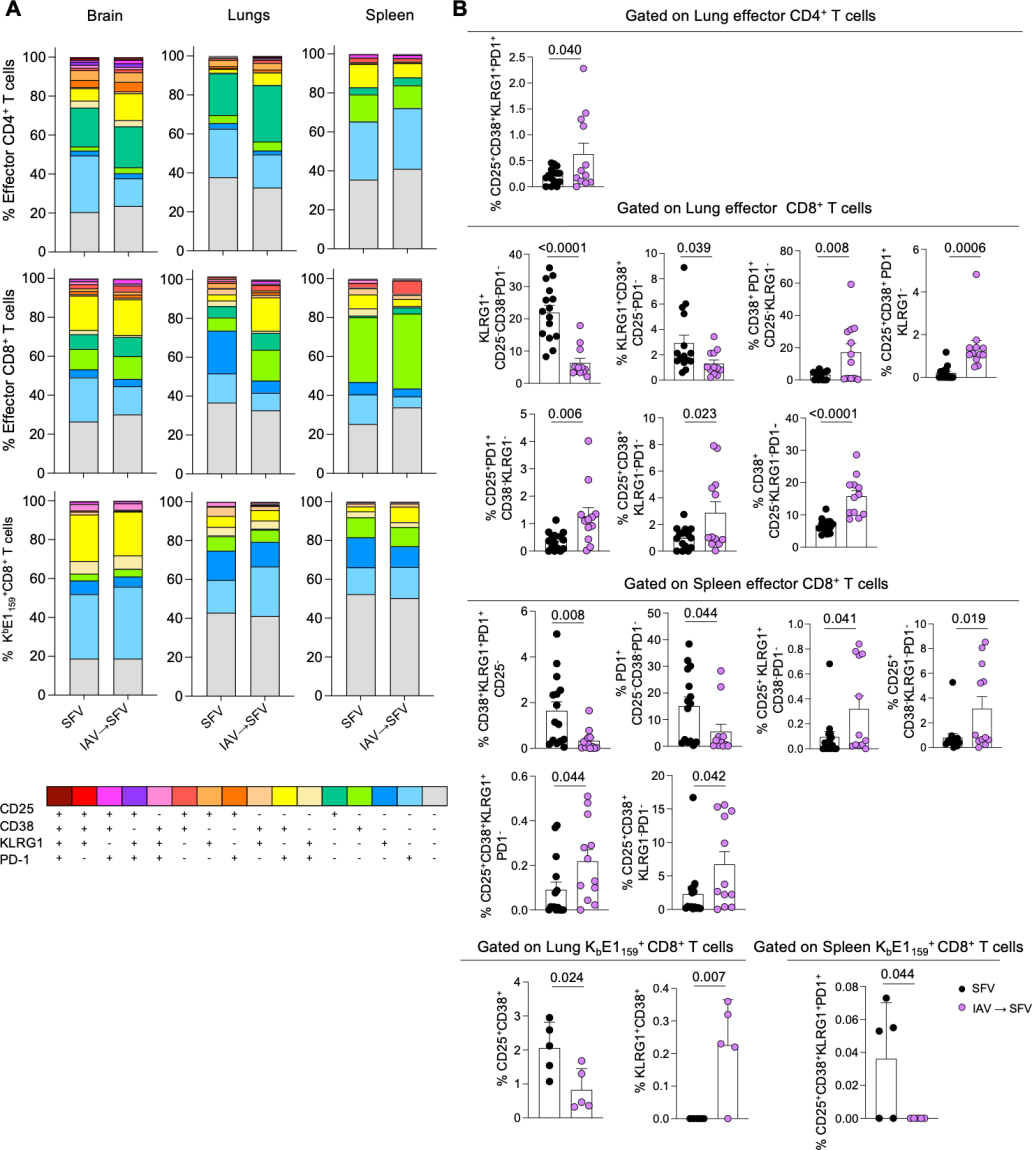
Activation profiles of CD8^+^ T cells in the lungs of SFV and IAV→SFV infected mice. (**A**) Stacked bar graphs depicting frequencies of activation marker combinations on effector CD4^+^ T cells (upper panel), effector CD8^+^ T cells (middle panel), and K^b^E1_159_^+^CD8^+^ T cells (bottom panel) on 7 dpi in SFV and IAV→SFV infected mice (*n* = 5–14, error bars represent SD for K^b^E1_159_^+^CD8^+^ T cells, SEM for effector CD4^+^ and effector CD8^+^ T cell populations). (**B**) Comparison of significantly different activation marker combinations in the lungs and spleens (*n* = 5–14, pooled data from three independent experiments, error bars represent SEM). Each symbol denotes an individual mouse. Significance was determined by unpaired Student’s *t* test. Data from 9 out of 14 SFV-only mice was used as a control in a previous publication, as indicated in source data (11).

To further characterize effector CD4^+^ T cells and CD8^+^ T cells, and SFV-specific CD8^+^ T cells, we defined key T cell activation markers (CD25, CD38, KLRG1, and PD-1) in the brains, lungs, and spleens of SFV and IAV→SFV infected mice (Fig. 4A). In the brains, activation phenotypes within effector T cell populations remained similar across infection types (Fig. 4A). However, in the lungs and spleens, we found distinct differences in the phenotypes of effector CD8^+^ T cells following SFV and IAV→SFV infections. The SFV-only infection induced CD8^+^ T cells with dominant phenotypes driven by the expression of KLRG-1, a marker of short-lived effector T cells (19), with significantly increased frequencies of KLRG1^+^, KLRG1^+^CD38^+^, KLRG1^+^CD38^+^PD1^+^ and KLRG1^+^CD38^+^CD25^+^ CD8^+^ T cell populations, compared to IAV→SFV infection (Fig. 4B). In contrast, IAV→SFV CD8^+^ T cell phenotypes had increased frequencies of CD38 and/or CD25 expression without expression of the KLRG1 short-lived effector marker, namely CD38^+^CD25^+^PD1^+^, CD38^+^CD25^+^, CD25^+^PD1^+^ and CD38^+^ CD8^+^ T cell populations. Analysis of SFV-specific CD8^+^ T cells demonstrated SFV-only infection to have higher frequency of CD25^+^CD38^+^ double positive phenotype, whereas IAV→SFV co-infection had higher KLRG1^+^CD38^+^ expression (Fig. 4B).

Overall, our data clearly indicate differential activation profiles of effector CD8^+^ T cells following single SFV infection or sequential IAV→SFV viral co-infection, most likely reflecting differential inflammatory and immune cell milieu.

### SFV and IAV→SFV mice have comparable resident CD8^+^ T cell memory pools

**Fig 5:**
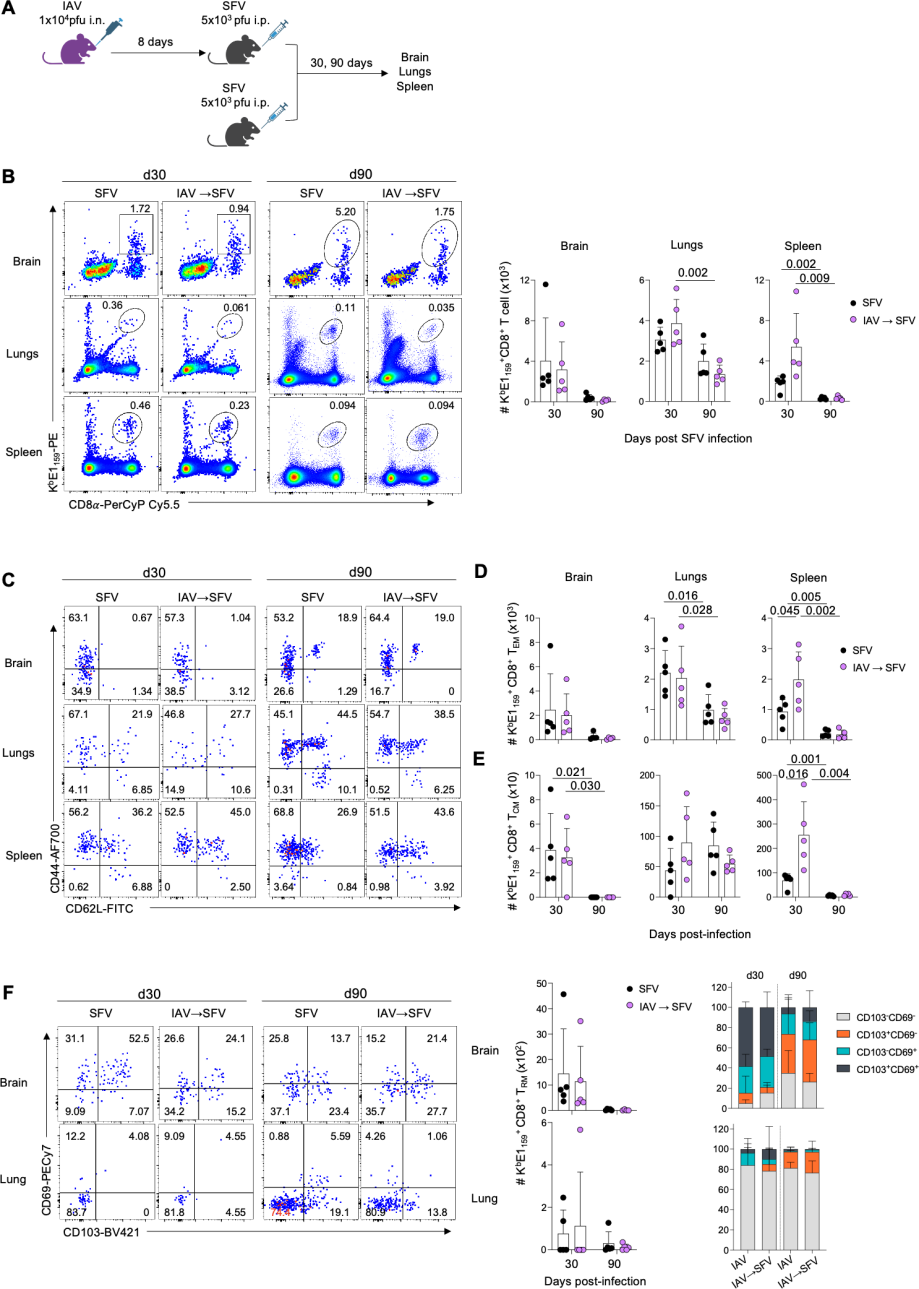
Numbers of long-term memory SFV-specific CD8^+^ T cells across different anatomical sites in SFV and IAV→SFV. (**A**) A group of mice were infected with 10^4^ pfu A/HKx31 (i.n.) followed 8 days later with 5 × 10^3^ pfu A7(74) SFV (i.p.). A second group of mice, received SFV-only. Brains, lungs, and spleens were analyzed on days 30 and 90. (**B**) Absolute numbers of SFV-specific CD8^+^ T cells directed at the K^b^E1_159_ epitope are shown across different anatomical sites in SFV and IAV→SFV infected mice at 30 and 90 dpi. Representative FACS plots shown for each group and tissue at different time points (*n* = 5, error bars represent SD). (**C–E**) Concatenated FACS plots (*n* = 5) shown for each group and tissue. Absolute numbers of SFV-specific T_EM_ and T_CM_ CD8^+^ T cells are shown for K^b^E1_159_^+^CD8^+^ T cell specificities in brains, lungs, and spleens of SFV and IAV→SFV infected mice at 30 and 90 dpi (*n* = 5, error bars represent SD). (**F**) Absolute numbers and frequencies of SFV-specific T_RM_ CD8^+^ T cells are shown for K^b^E1_159_^+^CD8^+^ T cell specificities in the brains and lungs of SFV and IAV→SFV infected mice at 30 and 90 dpi. Concatenated FACS plots (*n* = 5) shown for each group and tissue; (*n* = 5, error bars represent SD). Each symbol denotes an individual mouse, pooled data from two independent experiments,. Significance was determined by unpaired Student’s *t*-test.

IAV infection prior to SFV infection results in reduced levels of infectious SFV in the brain and a reduced inflammatory response (Fig. 1C, 2A, 3B and C). We next asked whether co-infection affects the generation of long-term resident CD8^+^ T cell memory (T_RM_), together with other CD8^+^ T cell memory populations; CD8^+^ T cell central (T_CM_) and CD8^+^ T cell effector (T_EM_) memory pools. The first experimental group of mice was infected i.n. with 10^4^ pfu IAV (×31) and 8 days later infected i.p with 5 × 10^3^ pfu SFV (A7(74)). The second experimental group was infected only with SFV. Brains, lungs, and spleens were analyzed 30 and 90 days after SFV infection (Fig. 5A).

SFV-specific (K^b^E1_159_) CD8^+^ T cells were detected across tissues in both groups of mice (Fig. 5B). At 90 dpi, SFV-specific CD8^+^ T cells in the lungs of the IAV→SFV group were significantly decreased compared to 30 dpi. Similarly in the spleens, numbers of SFV-specific CD8^+^ T cells were significantly reduced by 90 dpi in both SFV-only and IAV→SFV infection (Fig. 5B).

There were significantly lower numbers of T_EM_ in the lungs at 90 dpi compared to 30 dpi in both experimental groups (Fig. 5C and D), while the numbers of T_CM_ were comparable (Fig. 5E). Conversely, we found significantly lower numbers of T_CM_ in the brain at 90 dpi compared to 30 dpi in both infection types, but no statistical difference in numbers of T_EM_ (Fig. 5D and E). We also observed reduced numbers of T_EM_ and T_CM_ in the spleens at 90 dpi across both infection types. However, we observed that the IAV→SFV group in the spleens had significantly higher number of T_EM_ and T_CM_ at 30 dpi compared to SFV-only infection (Fig. 5C through E). At 90 dpi, we could still detect SFV-specific CD8^+^ T cell memory in both SFV-only and IAV→SFV co-infection across brains and lungs. However, we found no differences in numbers and proportions of SFV-specific T_RM_ across both time points and infection types (Fig. 5F).

Overall, our study provides evidence for protective immunity against SFV infection, stemming from reduced disease severity and inflammatory immune responses at the site of infection, that is, the brains, subsequently leading to decreased immune cell trafficking.

## DISCUSSION

Viral co-infections can substantially impact antiviral responses (8). We previously showed that SFV infection prior to IAV infection changes immune responses to IAV, resulting in altered T cell trafficking, prolonged IAV replication in the lungs, greater lung inflammation, and exacerbated lung pathology (11). The present study investigates the effect of a prior IAV infection on the course of an SFV infection. The results are quite different with reduced SFV replication and inflammation in the brain.

Both IAV and SFV viral strains used in our co-infection model are well-characterized and provide a robust model to study immune responses in the brains and lungs. The loss of up to 10% of initial body weight following SFV infection and up to 20% following IAV infection are consistent with our previous findings (20–22). Mice infected with IAV generally lose weight across the first 7 days and then start to recover. Inoculation with SFV at day 8 did not change this pattern and, despite the second infection, these co-infected mice gained weight. In mouse disease models, temporary weight loss generally reflects viral pathogenicity (23). The weight recovery in the IAV→SFV mice after SFV infection, a time at which SFV-only infected mice would be expected to lose weight, indicates that the clinical course and probably the underlying pathogenesis of the SFV infection was attenuated by the prior IAV infection. This pattern continues long term (Fig. S3A), where IAV→SFV co-infected mice continue to fare better than both IAV-only and SFV-only mice.

This was confirmed by lower titers of infectious SFV in the brains of IAV→SFV mice compared to SFV-only mice (Fig. 1C). Early in infection, days 3 and 5, levels of the chemokines CXCL1 and CCL2 were also lower in the brains of co-infected mice (Fig. 2B). Chemokines are released by virus infected cells, including brain cells such as neurons and astrocytes. It is likely that their lower titer in the IAV→SFV group resulted from the lower levels of brain virus in these mice. CXCL1 is important for transendothelial migration of neutrophils across the blood-brain barrier, and is upregulated by astrocytes and neurons in response to central nervous system (CNS) infection (12, 13, 24). CCL2 is associated with infiltration of inflammatory monocytes into the brain during acute viral infection (14, 15, 25, 26).

Consistent with the reduced virus titers and chemokine levels, the co-infected mice had lower levels of inflammatory cells in the brain at day 7 (Fig. 3A). However, it was not clear which cell populations were responsible for this difference since levels of neutrophils, macrophages, total CD8^+^ and CD4^+^ T cells, B cell, and antibody-secreting cells showed no significant differences (Fig. 3B and C). As reported in our previous studies, CD8^+^ T cells are particularly important in SFV brain pathology (27, 28) and they are the predominant brain T cell population in this study. Consistent with the lower virus titers, the numbers of total CD8^+^ T cells and activated CD8^+^ T cells (CD62L^lo^CD44^hi^) were significantly lower in the brains of the co-infected mice (Fig. 3D and E).

The lower brain virus titer does not appear to have resulted from lower systemic virus titers as the blood viremia in both groups at days 1 and 3 was comparable. SFV is highly sensitive to IFN and type I IFN is known to contribute to SFV viral clearance in many tissues including the brain (29, 30). IAV is a known inducer of type I IFN (31, 32) and these IFNs were present in the brain at low level 3 and 5 days after an IAV infection, peaking at day 7 and returning to baseline 14 days post-IAV infection (Fig. 2C). The x31 IAV strain used in this study is not known to be neurotropic but it is possible that the induction of type I IFN systemically by the IAV infection primed CNS antiviral responses (33). Given that SFV was inoculated into mice at day 8 post-IAV infection, when IFNα and IFNβ in the brain are at their peaks, it is likely that IFN activity is partially responsible for the reduced SFV brain infection and subsequent inflammation in the co-infected mice. In addition, it has been shown that the matrix (M) antigen is present in the brain of IAV-only infected mice (11), suggesting induction of antiviral responses against IAV antigen in the brain.

The situation in the lungs is different from that in the brain. In the co-infected mice, IAV would have replicated and established inflammatory and immune responses in the lungs for 8 days prior to SFV infection. SFV can replicate in many peripheral tissues including the lungs, but it is rapidly curtailed by the type I IFN response (29) and is generally undetectable by infectivity assays. Early in SFV infection (days 3 and 5), the levels of CXCL1 and CXCL10 were significantly lower in the lungs of the co-infected mice. This could have resulted from reduced SFV replication in the IAV-infected inflamed lungs (17, 34, 35). However, by day 7, cytokine levels were high in both experimental groups, and trended toward being higher in the co-infected mice. TNF was significantly higher in this group. The chemokine/cytokine data suggest that SFV established a strong response in the lungs of both groups of mice.

The analysis of inflammatory cell populations (Fig. 3) also supports a lung response to SFV infection. The higher numbers of total T and B cells in the co-infected lungs most likely reflect the preceding and ongoing IAV lung infection and associated inflammation. Such increase can again be associated with IAV clearance in the lungs.

Analysis of T cell activation markers indicated that bulk effector T cells and tetramer-positive CD8^+^ T cells in the lungs of IAV→SFV and SFV-only mice display distinct activation profiles. While CD8^+^ T cell populations from both groups express CD25, CD38, and PD-1, SFV infection *per se* induces high levels of KLRG1 expression, the marker of short-term-lived T cells (Fig. 4B). We observed that effector CD8^+^ T cells of SFV-only mice express KLRG1, and as mentioned previously SFV can replicate in extraneural tissues, perhaps leading to the upregulation of KLRG1 to activate CD8^+^ T cells and which later on, differentiate into short-lived effector CD8^+^ T cells (19). While KLRG1 is also a marker for T cell senescence in chronic viral infections (36–38), there is no suggestion in the literature that SFV can persist extraneurally. It is clear though that single SFV infection and sequential IAV→SFV viral co-infection induce differential activation profiles of effector CD8^+^ T cells, most likely reflecting differential inflammatory and immune cell milieu.

The long-term effects of IAV→SFV co-infection showed reduced numbers of SFV-specific CD8^+^ T cells from 30 to 90 dpi in the lungs and spleens, but also reduced number of tetramer-positive CD8^+^ T cells in SFV-only group. This is not surprising as the number of antigen-specific CD8^+^ T cells contracts by 90% after infection to leave a stable memory cell pool (39, 40). The total number of SFV-specific T_EM_ in the lungs and spleens also decreased 3 months after infection, as residual SFV antigen is mostly depleted from the draining lymph nodes, thereby any recently activated memory CD8^+^ T cell pools that contribute to the population of memory T cells subsequently decreased (41). While we expected to see an increase in the number of SFV-specific T_CM_ over time, we observed that these numbers were reduced instead, perhaps due to the contraction of total SFV-specific CD8^+^ T cells by 90 dpi. It appears that the total number of SFV-specific T_RM_ in the lungs and brain were similar across the infection types, meaning that both sequential and single infection could establish resident memory pools in the periphery and site of infection.

In conclusion, considering both this study and our previous study of IAV immunity in mice with or without a prior SFV infection (11), the order of sequential infection between two unrelated acute viruses can significantly alter the disease outcome. When mice were given SFV prior to IAV, it resulted in exacerbated respiratory disease (11). However, mice infected with IAV prior to SFV had milder neurotropic disease. Protective immunity by prior IAV infection has been documented previously whereby mice infected by IAV followed by SARS-CoV-2 recovered, whereas concurrent infection of IAV and SARS-CoV-2 presented more severe disease (42). In each case, the prior infection resulted in changes in levels of infectious virus, cytokines, chemokines, and the composition of the cellular immune response. Our study highlights the importance of characterizing immune responses across co-infection pairs, thereby preventing the generalization of co-infection outcomes and mechanisms.

## MATERIALS AND METHODS

### Viruses and virus infection

The influenza virus strain A/X31(H3N2) used in this study was grown in the allantoic cavity of 10-day-old embryonated chicken eggs in accordance with World Health Organization guidelines and ethics (WHO Flu AEC 27270). The titer of infectious virus was determined by plaque assay on monolayers of Madin Darby canine kidney cells, as described previously (43). The avirulent A7(74) strain of SFV was generated from plasmid pCMV-SFVA774-2SG provided by Professor Andres Merits (University of Tartu, Institute of Technology, Estonia). This plasmid expresses the viral genomic RNA under the control of a cytomegalovirus (CMV) promoter. SFV was grown in Baby Hamster Kidney fibroblasts (11, 42). A Vero cell plaque assay was used to titrate infectious SFV, as described previously (27).

Six- to eight-week-old mice were inoculated intraperitoneally (i.p.) with 5 × 10^3^ PFU of the avirulent A7(74) strain of SFV in 0.1 mL PBSA. For influenza virus infections, mice were lightly anesthetized with isoflurane (Veterinary Companies of Australia, Kings Park, NSW, Australia) using an anesthetic machine (Advanced Anaesthesia Specialists, Sydney, NSW, Australia) and infected by intranasal (i.n.) instillation of 10^4^ PFU of x31 (A/x31 (H3N2)) influenza virus in 30 µL phosphate-buffered saline (PBS). Naïve (uninfected) mice were also included as controls.

### Tissue sampling and single-cell preparation

Brains, lungs, and spleens were collected from mice at various time points after infection. Blood was first removed from the tissue vasculature by perfusion with 10 mL PBS through the left cardiac ventricle following terminal anesthesia. Brains were removed and processed for virus infectivity assay and chemokine/cytokine levels (half brain bisected sagittally along the midline), preparation of RNA for gene expression studies (half brain bisected sagittally along the midline), and analysis of cell infiltrates (entire brain). Single-cell suspensions were purified from the brains, lungs, and spleens. Brains were digested with 1,785 units/mL collagenase type III (Worthington Biochemical Corporation, Lakewood, NJ, USA) and 6 units/mL DNase I (Sigma-Aldrich, St. Louis, MO, USA) and leukocytes isolated by centrifugation on a Percoll gradient (70%, 37%, and 30% Percoll). Lungs were either homogenzed and centrifuged to obtain clarified supernatants to assay for viral titers/cytokine composition or enzymatically digested in 1 mg/mL collagenase III (Worthington) and 0.5 mg/mL DNase I (Sigma-Aldrich) before passing through cell sieves to obtain single-cell suspensions for analysis. Where necessary, cell suspensions from tissues were incubated with 0.15 M NH_4_Cl and 17 mM Tris-HCI at pH 7.2 for 5 min at 37°C to lyse red blood cells.

### Tetramer and phenotypic staining

MHC-I tetramers targeting the immunodominant epitope of the SFV E1 envelope protein (K^b^-E1_159-166_: TQFIFGPL) (11) were produced in-house and conjugated to streptavidin-PE (BD Biosciences, San Jose, CA, USA, 554061) or APC (BD Biosciences 554067) at 1:200 or 1:100 dilution respectively at room temperature for 1 h in the dark. Lymphocytes were stained with combinations of fluorochrome-conjugated antibodies: BD Biosciences: anti-CD8α-PerCyP Cy5.5 (53-67; 551162), anti-CD44-Alexa Flour 700 (1M7; 560567), anti-CD4-APC Cy7 (GK1.5; 552051), anti-TCRβ-BV711 (H57-597; 563135), anti-CD25-PECF594 (PC61; 562694), anti-CD45.1-FITC (A20; 561871), anti-CD62L-FITC (MEL-14; 561917), anti-CD45.1-PE (A20; 553776), anti-V2αTCR-FITC (B20.1; 553288), anti-CD45R-APCCy7 (RA3-6B2, 552094), anti-CD38-BV711 (90; 740697), anti-CD138-PE (281-2; 553714), anti-CD11c-FITC (HL3; 557400), anti-Gr1-FITC (RB6-8C5; 553126), anti-CD64-AF647 (X54-5/7.1; 558539), anti-Ly6C-AlexaFlour700 (AL-21; 561237), anti-CD11b-BV605 (M1/70; 563015), anti-CD45.2-BV711 (104; 563685), anti-CD11c-PE (HL3; 553802), anti-SigLecF-PECF594 (E50-2440; 562757), anti-Ly6G-PECy7 (1A8; 560601). BioLegend (San Diego, CA, USA): anti-CD62L-BV570 (MEL-14; 104433), anti-CD279-BV785 (29F.1A12; 135225), anti-CD38-PECy7 (90; 102718), anti-CD8α-BV510 (53–6.7; 100752), anti-CD103-BV421 (2E7; 121422), anti-CD69-PECy7 (H1.2F3; 104512), anti-CD62L-PECy7 (MEL-14; 104418), anti-GL7-PerCyPCy5.5 (GL7; 144610), anti-CD19-APC (6D5; 11512), anti-I-Ab-PacBlue (AF6-120.1, 116422), anti-IgD-PECy7 (11-26c.2a; 405720), anti-CD3-FITC (145-2C11; 100306), and anti-F4/80-FITC (RB6-8C5; 553126). Invitrogen eBiosciences (Waltham, Massachusetts, USA) (anti-KLRG1-FITC (2F1; 11-5893-82). Cell viability was determined by staining with either Live/Dead-Aqua 525 (L34966A, ThermoFisher, Waltham, Massachusetts, USA) or Live/Dead Fixable Near-IR (L10119, ThermoFisher). Cells were fixed with 1% paraformaldehyde before analysis by flow cytometry. Antibody staining was performed at 4°C in the dark. Samples were subsequently acquired on a BD LSR Fortessa (BD Biosciences) flow cytometer and data were analyzed by FlowJo Software (Tree Star Inc., USA). Gating strategies for flow cytometry data are described in Fig. S2 and S3D.

### Viable cell count

The number of viable cells per organ was calculated by diluting cell suspensions in 0.2% Trypan Blue and counting viable cells in a hemocytometer.

### Cytokine analyses

Cytokine and chemokine levels in lungs and brains homogenates were analyzed using the LEGENDPlex Multi-Analyte Flow Assay Kit (BioLegend) Mouse Anti-Virus Response Panel (13-plex) according to the manufacturers’ instructions.

### Statistical analyses

Statistical analyses were performed using the Student’s unpaired *t* test for comparisons between two groups, Tukey’s multiple comparison test, or Holm-Sidak’s multiple comparison test for comparisons among multiple groups within GraphPad Prism 10 software.

## Data Availability

The published article includes all data sets generated or analyzed during the study.

## SUPPLEMENTAL MATERIAL

Supplemental material jvi.02108-24-s0001.pdf Figures S1 to S3.

ASM does not own the copyrights to Supplemental Material that may be linked to, or accessed through, an article. The authors have granted ASM a non-exclusive, world-wide license to publish the Supplemental Material files. Please contact the corresponding author directly for reuse.
