# Supplementary material for: Prior influenza virus infection alleviates an arbovirus encephalitis by reducing viral titer, inflammation, and cellular infiltrates in the central nervous system
Source: J Virol. 2025 Jan 16;99(2):e02108-24. doi: 10.1128/jvi.02108-24 (PMC11852871; doi:10.1128/jvi.02108-24)
Supplement: Supplemental material — Figures S1 to S3. [file jvi.02108-24-s0001.pdf]

Supplemental Material

Prior influenza virus infection alleviates an arbovirus encephalitis by reducing viral titre, inflammation and cellular infiltrates in the central nervous system

Isabelle JH. Foo[1,2], Brendon Y. Chua[1], So Young Chang[1], Xiaoxiao Jia[1], Alice van der Eerden[1], John K. Fazakerley[1,2], Katherine Kedzierska[1*], Lukasz Kedzierski[1*]

[1]*Department of Microbiology and Immunology, The University of Melbourne, at the Peter Doherty Institute for Infection and Immunity, Melbourne, VIC, Australia*

[2]*Department of Veterinary Biosciences, Faculty of Science, University of Melbourne, Melbourne, VIC, Australia*

*Corresponding author
Email: lukaszk@unimelb.edu.au (LK), kkedz@unimelb.edu.au (KK)

Running Title: Prior influenza infection alleviates Semliki Forest Virus disease

SUPPLEMENTARY FIGURE LEGENDS

Supplementary Figure 1. (A) Cumulative concentrations of 10 cytokines and chemokines in the brains of IAV infected mice (n=5).

Supplementary Figure 2. Gating strategy for flow cytometric analysis of lymphocytes in the brain, lungs, and spleen. Antibodies against surface markers (see Methods section) were used in combination in 3 panels to gate on (**A)** myeloid cells, (**B)** B cells, and (**C)** T cells. Values indicate the percentage of gated or positive cells.

Supplementary Figure 3. (**A)** Weight loss of SFV, IAV, and IAV→SFV infected mice was monitored for 100 days (n= 5, error bars represent SD). Area under the curve (AUC) was determined by Ordinary one-way ANOVA with Holm-Sidak's multiple comparison test. Representative FACS plot of **(B)** effector ($CD44^+CD62L^{lo}$) $CD8^+$ (left) and **(C)** effector $CD4^+$ (right) T cells across different anatomical sites in IAV, SFV, and IAV→SFV infected mice. **(D)** Gating strategy for flow cytometric analysis of lymphocytes in the brain, lungs, and spleen at 30 and 90 dpi. Antibodies against surface markers (see Methods section) were used in combination to gate on SFV-specific $CD8^+$ T cells, SFV-specific effector, central, and resident memory $CD8^+$ T cells. Values indicate the percentage of gated or positive cells.

A

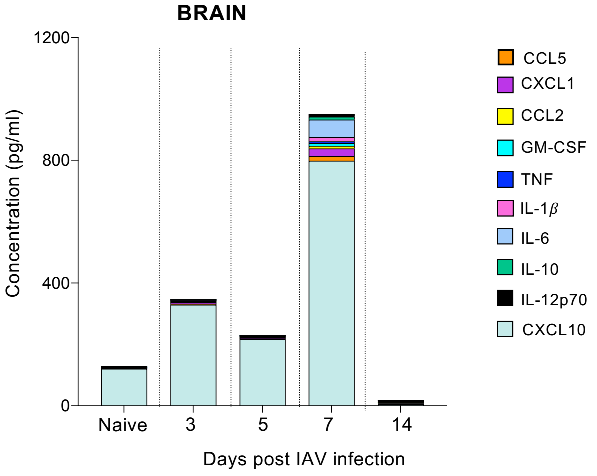

Supplementary Figure 1

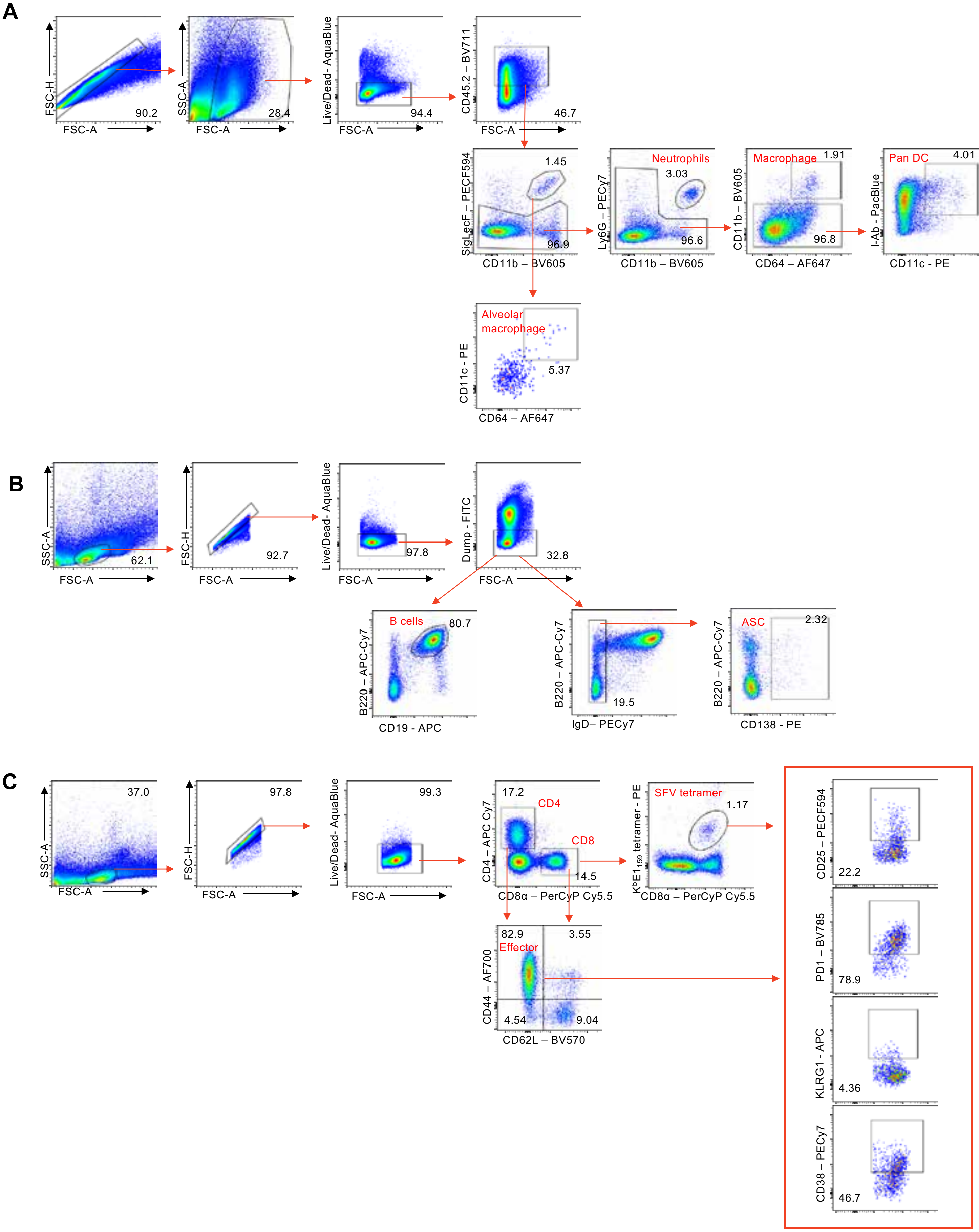

Supplementary Figure 2

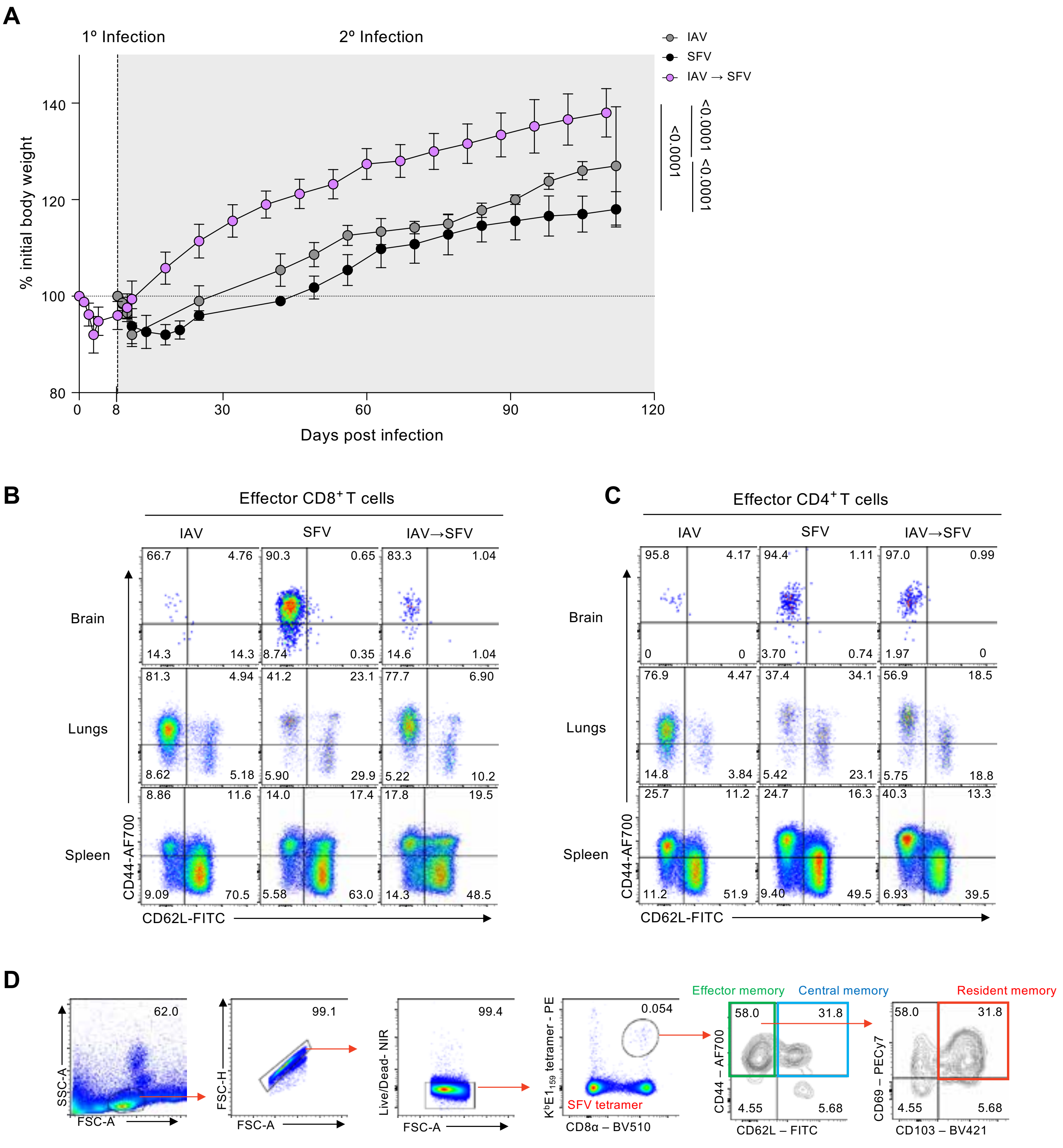

Supplementary Figure 3
